# The involvement of Canadian physicians in promoting and providing unproven and unapproved stem cell interventions

**DOI:** 10.1186/s12910-018-0273-6

**Published:** 2018-05-02

**Authors:** Ubaka Ogbogu, Jenny Du, Yonida Koukio

**Affiliations:** 1 Faculties of Law and Pharmacy & Pharmaceutical Sciences, University of Alberta, Edmonton, Alberta Canada; 2 Faculty of Law, University of Alberta, Edmonton, Alberta Canada; 3 Osgoode Professional Development, Osgoode Hall Law School, York University, Toronto, Canada

**Keywords:** Stem cell interventions (SCIs), Stem cell, Stem cell treatment, False advertising, Canada, Physicians, Unproven treatments, Stem cell tourism

## Abstract

**Background:**

Direct to consumer offerings of unproven stem cell interventions (SCIs) is a pressing scientific and policy issue. According to media reports, providers of SCIs have emerged in Canada. This study provides the first systematic scan of Canadian providers and associated trends and claims.

**Methods:**

The study sample consisted of 15 websites retrieved from a Google™ keyword search. The websites were assessed by a rater using a peer-reviewed coding frame that queried treatment location, stem cell offerings, treatment claims, supporting evidence, and legal and regulatory compliance. A second rater reviewed a subset of the websites for purposes of inter-rater reliability. Disagreements between raters were resolved by consensus. Data collected by the raters was analyzed in SPSS.

**Results:**

Physicians are the dominant treatment providers in Canada. Providers operate in urban and semi-urban areas in the most populous provinces. SCIs provided are mainly autologous adult stem cells for multiple conditions including musculoskeletal disorders, spinal cord injury (SCI) and diabetes. Efficacy and benefits of treatment are prominently and positively portrayed, while risks are not mentioned or portrayed as trivial. Regulatory concerns are not discussed.

**Conclusions:**

The involvement of physicians in promoting and providing unproven and unapproved SCIs raises significant ethical, legal and regulatory concerns. Treatment claims and trends appear to contravene applicable professional standards, statutory obligations, and consumer protection laws. While the number of providers observed is still marginal, urgent and proactive regulatory response is needed to prevent proliferation of a potentially exploitative and harmful market for unproven SCIs in Canada.

**Electronic supplementary material:**

The online version of this article (10.1186/s12910-018-0273-6) contains supplementary material, which is available to authorized users.

## Background

Stem cell interventions (SCIs) offered to the public in advance of clinical testing and regulatory approval is a pressing scientific, health policy and patient/public welfare issue [1–7]. Despite efforts to stem the phenomenon, providers have proliferated worldwide, mainly due to growing public demand for SCIs and weak or supportive regulations [8–12]. To date, the only SCIs approved for clinical use by Health Canada are hematopoietic stem cell transplants from bone marrow, umbilical cord and peripheral blood for treatment of blood-based cancers, blood disorders and solid tumour cancers [13]. Health Canada has also issued conditional approval for Prochymal, a mesenchymal stem cell product used to treat Graft-Versus-Host Disease in pediatric patients [13]. Media reports suggest that providers of SCIs that have not been approved by Health Canada have recently emerged in Canada [14, 15]. This is a notable shift from trends observed in previous studies, which suggest that Canada is a jurisdiction of patients and end users who typically seek treatment with unproven SCIs abroad [9, 10]. This paper reports the results of the first systematic study of Canadian providers of putative, unapproved and scientifically unproven SCIs. Although the study assessed online presence, claims and offerings of Canadian providers, we focus specifically on the involvement of Canadian physicians. Our findings show that Canadian physicians are the dominant players in the online promotion and provision of unproven and unapproved SCIs and suggest an urgent need for policy action and response from both within and outside the profession.

## Methods

The study design is based on a well-established peer-reviewed content analysis method that is used in studies of health information online [9–11, 16–18]. The study sample consisted of 15 websites retrieved from a Google™ search for “stem cell therapy OR clinic OR treatment OR hospital.” Two searches were conducted; the first was limited to Canada, and the second to each province and territory in Canada. We retrieved the first 20 pages of each search. The initial sample consisted of 61 websites. We excluded websites promoting or providing SCIs approved by Health Canada, or cell-based treatments that do not contain stem cells (e.g. platelet-rich plasma therapy), or treatments or services for animals exclusively, or with limited or no information, or that offered products only and not clinical procedures, or with a principal place of business outside of Canada. The final sample included only websites that promote or provide clinical interventions. The websites were analyzed by two raters using a peer-reviewed coding frame (see Additional file 1) that queried provider information, location, offerings, clinical procedures, and treatment claims. The first rater analyzed the full study sample, while the second rater reviewed a subset for purposes of inter-rater reliability. Rater observations were recorded on an excel spreadsheet (see Additional file 2) using values assigned to each variable in the coding frame. Disagreements between raters were resolved by consensus. The initial analysis was conducted between August 28, 2017 and September 1, 2017. The complete study data was analyzed in SPSS, a statistical analysis software that can be used to perform descriptive statistics, including frequencies and cross-tabulations, on assigned values, such as those recorded by the raters in the excel spreadsheet. An additional file shows the results of the SPSS analysis (see Additional file 3).

## Results

**Fig. 1:**
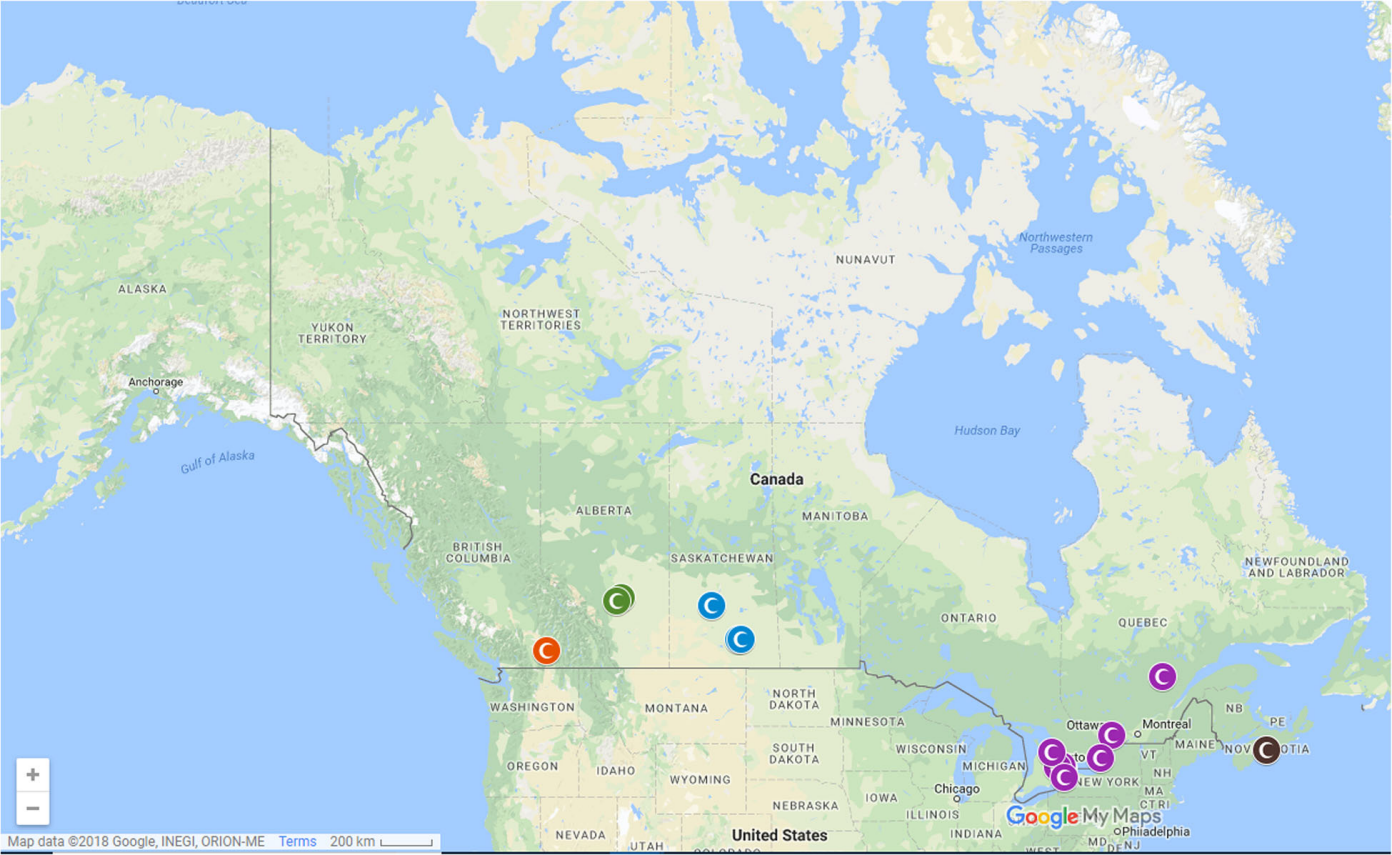
Map of locations, by province, of providers of stem cell interventions included in the study Source: Google Maps™. Per Google™ use and attribution guidelines (https://www.google.com/permissions/geoguidelines.html), the necessary attribution is automatically embedded in the image (in the bottom left corner). Google™ permits non-commercial use of printed Google Maps™ content in journals

**Fig. 2:**
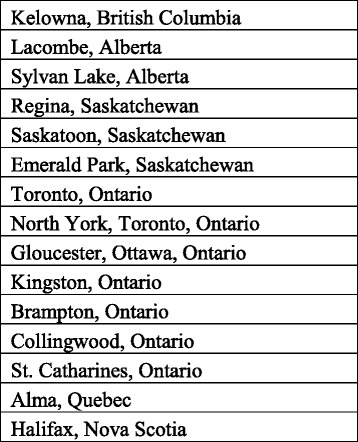
Table of location, by place, of providers of stem cell interventions included in the study

Twelve (12) of the 15 websites in the study feature SCIs provided by physicians. The providers operate in urban and semi-urban areas in Ontario (7 providers), Saskatchewan (3 providers), Alberta (2 providers), British Columbia (1 provider), Quebec (1 provider) and Nova Scotia (1 provider) (see Figs. 1 and 2). We did not find any treatment providers in Manitoba, the rest of Atlantic Canada, and the Territories. Thirteen (13) providers use adult stem cells for the clinical interventions promoted on their websites, while 2 providers do not specify the type of stem cells used. Eleven (11) providers offer autologous transplants, while 4 do not specify transplant source. Of the 10 providers that specify stem cell lineage, 6 offer mesenchymal stem cells and 4 provide both haematopoietic and mesenchymal stem cells. Bone marrow was the most common source of stem cells (6 providers), followed by fat (3 providers) and teeth (1 provider). Three (3) providers use stem cells from multiple sources, mainly bone marrow and fat. The same number of providers (3) provide treatments for multiple conditions, including musculoskeletal disorders, spinal cord injuries, diabetes, multiple sclerosis, and skin diseases. Six (6) providers offer treatments for arthritis alone, and 7 providers do not mention or discuss conditions treated on their websites. Two (2) providers offer SCIs for aging, while 5 providers promote SCIs for general stress, fatigue, and cosmetic or health enhancement.

SCIs are portrayed as revolutionary (i.e. a marked departure from current therapies) and as clinically well-established and routine by all but one provider. None specify or discuss procedures for processing stem cells used or for quality assurance. Eight (8) providers mention or discuss treatment efficacy on their websites. Among these providers, 3 do not provide any support for claims regarding efficacy, while 4 cite either patient testimonials or claims about successful number of cases treated. The remaining provider references published scientific articles to support efficacy claims. Twelve (12) providers discuss treatment benefits, including improvement in disease state (10 providers), physical rejuvenation (improved vitality, better sleep, etc.) (1 provider) and cosmetic enhancement (1 provider). SCIs are portrayed as generally safe by 5 providers. Treatment risks are not mentioned or discussed by 10 providers. Four (4) providers discussed risks relating to impure transplants, such as infections, and minor side effects such as fever, headaches and post-treatment discomfort, while one provider does not specify treatment risk. Three (3) providers present the risks mentioned or specified as very unlikely, while two providers present associated risks as possible. Only 3 of the 15 providers mention or discuss laws and regulations relating to stem cells on their websites. Cost of treatment is mentioned or discussed by only one provider, amounting to $300 for the “initial assessment”, $2000 for the initial injection, and $100 “for each additional site.”

## Discussion

The study indicates that physicians dominate the emerging Canadian market for SCIs that are neither scientifically established nor approved for clinical use by Health Canada. While no studies have found a similar trend in or reached a similar conclusion for other jurisdictions, the involvement of physicians in promoting and providing unproven and unapproved SCIs has been observed in the United States (U.S.) [11] and Thailand [19]. In a 2009 review, Kiatpongsan and Sipp note, for example, that TheraVitae, a clinic providing unproven SCIs in Thailand, boasts “an impressive list of Thai physicians, including the…presidents of the Thai Heart Association and the Thai Atherosclerosis Society” ([19] p. 1564). In a seminal study of the U.S. context, Turner and Knoepfler observe that regulatory inaction may have “emboldened entrepreneurial physicians” to actively promote stem cell products in the U.S. ([11] p. 157).

Physicians, per legal, ethical and professional standards, are expected to promote and provide therapies that are backed by scientific evidence of safety and efficacy generated from clinical trials, and that are approved for clinical use. Offering SCIs in advance of clinical evidence of safety and efficacy, and absent regulatory approval, potentially violates applicable standards of care [20]. It also undermines efforts – professional, regulatory, societal or otherwise – to foster evidence-informed health care that balances innovation with responsible and ethical health care delivery [21, 22].

Regulatory response to these potential effects and violations is seriously lacking in Canada. While Health Canada has investigated some clinics [23], neither Health Canada nor the provincial colleges of physicians and surgeons have developed or adopted policies or strategies for dealing with the proliferation of unapproved SCIs [14]. By contrast, the U.S. Food and Drug Administration (FDA) recently announced a regulatory scheme that will require all human cells, tissues, and cellular and tissue-based products (HCT/P) to undergo full pre-market regulatory approval as investigational new drugs prior to clinical use or marketing [24]. The FDA also requires appropriate regulatory submissions for, and prior to, clinical trials, marketing, or routine clinical use [24]. HCT/P include haematopoietic and mesenchymal stem cells, which (this study and others have observed) are presently promoted by providers of unproven SCIs as ready for and used in routine clinical procedures. While it is not yet clear how the FDA plans to enforce this policy, it has established an expedited approval process for products designated as Regenerative Medicine Advanced Therapy (RMAT) [24]. The RMAT designation, as defined in the 21st Century Cures Act of 2016, applies to regenerative medicine therapies, including HCT/P, that are intended to treat or address a serious or life-threatening disease or condition, and for which there is preliminary clinical evidence of potential to address unmet medical needs [24, 25]. The RMAT expedited approval process aims to shorten the timelines for the development of cutting-edge HCT/P while ensuring that products that enter the market or clinic are backed by preliminary evidence of efficacy. The European Union (EU) has established a similar designation, known as advanced therapy medicinal products (ATMPs), for therapies for human use that are based on cells, genes or tissue engineering [26, 27]. ATMPs are regulated centrally through the European Medicines Agency and receive EU-wide market authorization [27]. The EU also has an accelerated assessment process for ATMPs [28]. However, the EU process merely prioritizes and reduces the timeframe for review of products that are of major interest for public health and therapeutic innovation and does not vary or reduce the evidentiary requirements for market authorization.

The practices and trends observed in our study also appear inconsistent with existing modes of delivering investigational therapies to patients. While investigational therapies may be offered to patients through compassionate or expanded access programs, access to such programs is regulated and available to a limited number of patients who meet the qualification criteria. Compassionate and expanded access programs are also evidence-based and are not intended to serve as “carte blanche” for routine clinical administration of unproven therapies. For example, EU regulations allow member states to permit compassionate use of “investigational ATMPs…[to address] life threatening unmet medical need” ([29] p. 17). Compassionate use is also possible under a “hospital-exemption” provision, which allows member states to authorize individualized ATMPs that are used in a hospital setting to treat an individual patient [29]. ATMPs for compassionate use must comply with traceability, pharmacovigilance and quality standards, but may be approved based on less comprehensive safety evidence than normally required [27, 29]. Elsanhoury and colleagues have argued, and we agree, that EU compassionate use programs and hospital exemptions are not intended to circumvent or obviate the need for “prospective product development and high-evidence clinical trials”, or to incentivize or facilitate clinical uses of unproven stem cell therapies ([29] p. 17). Australia also has a “medical practice exemption” that permits unapproved uses — presumably including compassionate uses — of stem cells obtained from a patient to treat a single indication in a single course of treatment of that patient [30]. However, upcoming changes to Australian regulations will tighten the criteria for the medical practice exemption and introduce regulations for autologous cell and tissue products that are minimally manipulated, manufactured and used outside hospital settings, and intended for homologous use in a patient under the care of a medical or dental practitioner [30]. The latter changes aim, in part, to address the “growing global concern with direct to consumer advertising of unproven autologous stem cell interventions” [30]. Health Canada, unlike its EU and Australian counterparts, has not issued any guidance on compassionate use of SCIs. In any event, we did not find any evidence that the SCIs in our study were offered as part of compassionate or expanded access programs. Rather, most offerings were portrayed as clinically well-established or ready for routine application.

The involvement of physicians in providing unproven and unapproved SCIs is also problematic from a consumer protection standpoint [5, 31]. Physicians are legally obligated to avoid misleading the public regarding the benefits of the therapies they provide. The broad and unsubstantiated claims on efficacy, benefit and safety observed in the study most likely violates consumer protection laws. The role of consumer protection law in combating the promotion and marketing of unproven and unapproved SCIs is well-canvassed in the literature [5, 31], and need not be repeated here. Generally, consumer protection legal instruments in Canada, Australia, and the U.S., among others, contain provisions that directly address the marketing practices of providers of unproven SCIs [5, 31], but enforcement is hampered by “systemic factors” [31] such as resource constraints, lack of effective strategies for policing cross-border online marketing, and overreliance on consumer complaints [5, 31].

The physicians in our study maintain a presence online and in urban and semi-urban areas in Canada’s most populous provinces. This suggests efforts to provide accessible service and maximize potential clientele. At the same time, these physicians seem to be operating in places where they are more likely to attract notice, including from critics, media and regulators.

The findings regarding portrayal of benefits, risks and efficacy of treatments provided align with previous studies [8–12]. Generally, efficacy and benefits are exaggerated, and risks are either not mentioned or downplayed. Claims are rarely supported, and where support exists, it is mainly of the non-credible variety.

The study has two main limitations. First, the study results and analysis are not generalizable, as the number of websites surveyed is very limited. The study sample may not be representative of the actual number of providers in Canada. Second, the study methods are subject to interpretative biases commonly associated with content analysis. As such, the study should not be interpreted as establishing definitive trends, practices, or events.

## Conclusions

Our findings suggest a need for timely and proactive intervention to prevent the normalization of questionable clinical practices. At a minimum, professional Colleges and health regulators ought to investigate the issue and assess the legitimacy of associated trends and practices. A first step in this direction might be to implement governance strategies that emerged from previous initiatives by Canadian medical societies [32].

## Additional files

Additional file 1: Coding frame used to collect the study data. (DOCX 37 kb)

Additional file 2: Study raw data file. (XLSX 15 kb)

Additional file 3: File showing outputs of statistical analysis (frequencies) performed in SPSS. (PDF 92 kb)

## Abbreviations

ATMPs: Advanced Therapy Medicinal Products
HCT/P: Human Cells, Tissues, and Cellular and Tissue-Based Products
RMAT: Regenerative Medicine Advanced Therapy
SCIs: Stem Cell Interventions
