# Supplementary material for: The involvement of Canadian physicians in promoting and providing unproven and unapproved stem cell interventions
Source: BMC Med Ethics. 2018 May 2;19:32. doi: 10.1186/s12910-018-0273-6 (PMC5930514; doi:10.1186/s12910-018-0273-6)
Supplement: Supplementary file 1 — Coding frame used to collect the study data. (DOCX 37 kb) [file 12910_2018_273_MOESM1_ESM.docx]

**The Involvement of Canadian Physicians in Promoting and Providing Unproven and Unapproved Stem Cell Interventions**

**Coding Frame**

1. Basic Information
   1. Name of organization
   2. Website URL
   3. Date website coded
   4. Primary/main clinic location within Canada
      - N/A – 99
      - Unspecified – 0
      - British Columbia – 1
      - Alberta – 2
      - Saskatchewan – 3
      - Manitoba – 4
      - Ontario – 5
      - Quebec – 6
      - Newfoundland and Labrador – 7
      - New Brunswick – 8
      - Prince Edward Island – 9
      - Nova Scotia – 10
      - Territory: Yukon – 11
      - Territory: Northwest Territories – 12
      - Territory: Nunavut – 13
   5. Whether the company has a connection to a foreign provider
      - N/A, same as above – 99
      - Unspecified – 0
      - Australia – 1
      - South Africa – 2
      - Turks and Caicos – 3
      - USA – 4
      - Multiple locations (specify) – 5
      - Other (specify) – 6
2. Treatment Information
   1. Stem cell offerings
      - Unspecified - 0
      - Embryonic stem cells (eSC) – 1
      - Fetal stem cells (fSC) – 2
      - Adult stem cells (aSC) – 3
      - Cord blood cells (cbSC) – 4
      - iPS cells – 5
      - Stem cell adjuncts (e.g.: telomerase) – 6
      - Other (specify) – 7
      - Multiple sources (specify) – 8
      - No stem cell therapy offered, but other treatment(s) promoted as stem cell therapy e.g. platelet-rich plasma therapy (specify) – 9
   2. Is stem cell therapy autologous, allogeneic, or xenogeneic?
      - N/A, no stem cells provided – 99
      - Unsure/unspecified - 0
      - Autologous– 1
      - Allogeneic – 2
      - Xenogeneic – 3
      - Multiple (specify) – 4
   3. Is the stem cell lineage specified?
      - N/A – 99
      - Not mentioned – 0
      - No, the cells are pluripotent – 1
      - Yes, haematopoietic – 2
      - Yes, mesenchymal – 3
      - Multiple (specify) – 4
   4. Stem cell source
      - N/A – 99
      - Unspecified – 0
      - Yes, from fat – 1
      - Yes, from bone marrow – 2
      - Yes, from peripheral or cord blood – 3
      - Yes, from aborted foetuses – 4
      - Yes, other (specify) – 5
      - Yes, from multiple sources (specify) – 6
   5. Are transplant processing or quality assurance procedures specified?
      - N/A – 99
      - No – 0
      - Yes – 1
   6. Transplantation procedure
      - N/A – 99
      - Unspecified - 0
      - Yes, intravenous injection – 1
      - Yes, subcutaneous injection – 2
      - Yes, by mouth – 3
      - Yes, surgical transplantation to organ of interest – 4
      - Other (specify) – 5
      - Yes, multiple procedures (specify) – 6
   7. Is immune rejection of the graft addressed in the treatment protocol?
      - N/A – 99
      - No (not addressed or no risk of immune rejection) - 0
      - Yes, by HLA-matching - 2
      - Yes, by immunosuppression – 3
      - Yes, by other means (specify) – 4
      - Yes, by multiple means (specify) – 5
   8. The treatment is portrayed as being
      - N/A, no longer treatment – 0
      - Experimental - 1
      - Ready for routine clinical application – 2
      - Clinically well-established – 3
   9. Is there mention of clinical trials conducted at clinic?
      - Not mentioned - 0
      - Yes, ongoing clinical trials – 1
      - Yes, completed clinical trials – 2
   10. Treatment cost
       - Unmentioned - 0
       - Mentioned, figures or ranges provided - 1
   11. If cost figures or ranges are provided, specify.
3. Stem cell science
   1. How the treatment works
      - No explanation - 0
      - Don’t know, exactly - 1
      - Stem cells migrate to pathology - 2
      - Stem cells replace damaged or depleted tissue - 3
      - Stem cells generate local environmental effects (e.g.: growth factor production) – 4
      - Stem cells fuse with existing cells – 5
   2. Stem cells and disease: Site portrays a deficiency in a finite pool of stem cells as a major mechanism or contributor to disease.
      - Not at all – 1
      - Somewhat - 2
      - Prominently - 3
   3. Is the treatment is portrayed as revolutionary, i.e.: a marked departure from current therapies?
      - Not at all – 1
      - Somewhat - 2
      - Prominently - 3
   4. Is the treatment portrayed as the clinical application of routine or familiar procedures?
      - Not at all – 1
      - Somewhat - 2
      - Prominently - 3
   5. The site appeals to holistic, natural, or magical powers of renewal
      - Not at all – 1
      - Somewhat – 2
      - Prominently – 3
   6. The site appeals to the scientific deconstruction of health and illness (e.g.: by emphasizing the technical complexity of treatment)
      - Not at all – 1
      - Somewhat – 2
      - Prominently - 3
   7. Knowledge is portrayed as being incomplete or uncertain
      - Not at all – 1
      - Somewhat – 2
      - Prominently - 3
   8. Knowledge is portrayed as being advanced or certain
      - Not at all – 1
      - Somewhat – 2
      - Prominently - 3
   9. Claims are substantiated *mainly* by ...
      - No claims, N/A – 0
      - Expert testimonial - 1
      - Expert consensus, experience - 2
      - Participation in a local regulatory regime - 3
      - Professional or academic institutions - 4
      - Anecdotes or the clinic’s previous experience with the therapy -5
      - Patient testimonials - 6
      - Lay literature (e.g.: news articles) - 7
      - Case reports - 8
      - Abstracts or presentations at conferences or meetings - 9
      - Published works (peer-reviewed) – 10
      - Published works (not peer-reviewed) – 11
4. Indications / Associated Conditions
   1. Manner of presentation
      - None are presented. - 0
      - Treatable diseases may be *inferred* from the clinic’s case reports, anecdotes, news reports, or patient testimonials – 1
      - The site lists treatable or previously treated diseases – 2
   2. Indeterminate vocabulary

(e.g.: “... and other conditions” or “Indications include ...”)

- - - No / not applicable - 0
    - Yes - 1
  1. Associated diseases
     - None - 0
     - Parkinson’s – 1
     - Alzheimer’s – 2
     - Diabetes - 3
     - Heart Disease - 4
     - Multiple Sclerosis (MS) – 5
     - Arthritis – 6
     - Blood cancers and diseases – 7
     - Skin disease/conditions – 8
     - Vascular disease – 9
     - Spinal cord injury – 10
     - Multiple (specify) – 11
  2. Is “aging” an indication?
     - No - 0
     - Yes - 1
  3. Are cosmetic or lifestyle enhancement purposes given as indications?
     - None - 0
     - Cosmetic - 1
     - General stress or fatigue - 2
     - Desire for health-enhancement, otherwise healthy - 3
  4. Are any conditions or patients excluded?
     - No – 0
     - Yes, those who don’t fit listed disease profile – 1
     - Yes, because the doctors think it will not improve their particular condition – 2
     - Yes, because there is some condition of the patients that would preclude stem cell implant, such as recent cancer or HIV or Hepatitis B and C – 3
     - Yes, other – 4

1. Efficacy of treatment
   1. Does website mention/discuss efficacy of treatment?
      - No – 0
      - Yes – 1
   2. If yes, is any evidence for efficacy provided?
      - N/A – 99
      - No evidence provided (just stated as effective) – 0
      - Claims about # (or %) of successful, safe treatments – 1
      - Patient testimonials – 2
      - Case studies conducted at clinic – 3
      - Published scientific articles – 4
      - Expert/doctor testimonial – 5
2. Benefits of treatment
   1. Manner of presentation
      - None are presented - 0
      - Benefits may be *inferred* from the clinic’s case reports, anecdotes, news reports, or patient testimonials – 1
      - The site lists previous or potential benefits – 2
   2. Treatment is portrayed to be generally beneficial
      - N/A, no discussion of benefits – 99
      - No - 0
      - Yes - 1
   3. Associated benefits include
      - No benefits are mentioned - 0
      - Improvement in disease state - 1
      - Physical rejuvenation (e.g.: improved vitality, better sleep) - 2
      - Mental rejuvenation (e.g.: improved cognition) - 3
      - Cosmetic enhancement - 4
      - Longer life – 5
   4. Likelihood of benefits
      - No benefits are presented – 0
      - Benefits are presented, but likelihood is not quantified - 1
      - Very unlikely (e.g.: rare) – 2
      - Unlikely - 3
      - Possible – 4
      - Likely – 5
      - Very likely (e.g.: certain) – 6
3. Risks of treatment
   1. Manner of presentation
      - None are presented. – 0
      - Risks may be *inferred* from the clinic’s case reports, anecdotes, news reports, patient testimonials – 1
      - The site lists previous or potential risks – 2
      - Risks may be *inferred* from the clinic’s disclaimer (about the risks of treatment) – 3
   2. Treatment is portrayed to be generally safe
      - N/A, no discussion – 99
      - No - 0
      - Yes - 1
   3. Associated risks include
      - No risks are presented – 0
      - Deterioration of disease state – 1
      - Immune rejection of stem cell graft – 2
      - Risks related to impure transplant materials (e.g.: infection) – 3
      - Surgical risks (e.g.: anaesthesia, infection) – 4
      - Graft versus host disease (GvHD) – 5
      - Teratoma or other cancer – 6
      - Minor side effects (e.g., fever, headache) – 7
      - Multiple (specify) – 8
      - Risks are mentioned but not specified – 9
   4. Likelihood of risks
      - No risks are presented – 0
      - Risks are presented, but likelihood is not quantified - 1
      - Very unlikely (e.g.: rare) – 2
      - Unlikely – 3
      - Possible – 4
      - Likely – 5
      - Very likely (e.g.: certain) – 6
4. Ethical issues
   1. Are social/ethical issues mentioned?
      - None – 0
      - Issues related to the source of transplantation
      - material (e.g.: moral status of the embryo) – 1
      - Issues related to the provision of unproven technologies – 2
      - Issues related to accessibility of health care – 3
   2. Relevancy
      - No social/ethical issues are mentioned – 0
      - Issues are irrelevant to the services provided – 1
      - Issues may or may not be relevant to the services provided – 2
      - Issues are relevant to the services provided – 3
   3. Has treatment received any official approvals?
      - No/Not mentioned – 0
      - Ethics approval – 1
      - Regulatory approval – 2
      - Complies with UNESCO guidelines – 3
5. Website aesthetics and intended audience
   1. This website appears to target
      - Patients – 1
      - Healthy individuals – 2
      - The general public – 3
      - Health professionals – 4
      - Policy-makers / health regulators – 5
      - Specific group of people, e.g., celebrities, cancer patients (specify) – 6
   2. Website appeal – the website appeals mainly through
      - Cosmesis – 1
      - Curiosity – 2
      - Desperation – 3
      - Disease prevention – 4
      - Health-enhancement (or improvement of disease state) – 5
      - Holism – 6
      - Normalization – 7
      - Technophilia (i.e.: a strong enthusiasm for technology) – 8
      - Legality/validity of operation – 9
      - Alternative to restrictive regulatory environment in home country – 10
   3. Visual style – the website is *mainly* ...
      - Under construction, not graphic or textual at this point – 0
      - Graphically oriented – the website features prominent decorative graphics and multimedia gadgets – 1
      - Text oriented – the website features prominent amounts of text – 2
      - Separate graphically oriented and text-oriented pages are available – 3
   4. Language – the website’s language is *mainly*
      - Under construction, very little text/information available – 0
      - Easily interpreted by lay viewers – 1
      - A mix of technical and easily interpreted passages – 2
      - Technical – 3
      - Separate lay and technical pages are available – 4
   5. Disclaimers/Legality – the website provides
      - No disclaimers mentioned – 0
      - Disclaimer about experimental nature of treatment – 1
      - Disclaimer that treatment is not FDA approved – 2
      - Explains that treatment is legal in country/location where it is provided – 3
      - Bypasses health regulatory barriers in all countries – 4
      - Other – 5
6. Other services available at clinic
   - - No mention of other services – 0
     - Physical therapy – 1
     - Occupational therapy – 2
     - Counselling – 3
     - Diagnostic imaging (e.g., MRI) – 4
     - Liberation treatment (CCSVI) – 5
     - CAM treatments – 6
     - Chinese medicine – 7
     - Nutritional counseling – 8
     - Other (specify) – 9
     - Multiple (specify) – 10
7. Please provide any other information that may seem relevant, or provides quotations of language used.
8. Does the website mention or discuss Canadian law/regulations relating to stem cells, embryonic stem cells or iPSCs?

- No – 0
- Yes – 1
