# Supplementary material for: The involvement of Canadian physicians in promoting and providing unproven and unapproved stem cell interventions
Source: BMC Med Ethics. 2018 May 2;19:32. doi: 10.1186/s12910-018-0273-6 (PMC5930514; doi:10.1186/s12910-018-0273-6)
Supplement: Supplementary file 3 — File showing outputs of statistical analysis (frequencies) performed in SPSS. (PDF 92 kb) [file 12910_2018_273_MOESM3_ESM.pdf]

```
 GET
  FILE='C:
\Users\uogbo\Dropbox\IMPACT GRANT\rescclinicstudycodingframe\Untitled2R.sav'.
DATASET NAME DataSet1 WINDOW=FRONT.
FREQUENCIES VARIABLES=Clinic# @1a.Nameofoganization @1b.WebsiteURL @1c.
Datewebsitecoded
    @1d.PrimarymaincliniclocationwithinCanada @1e.
Whetherthecompanyhasaconnectiontoaforeignprovider
    @2a.Stemcellofferings @2b.
Isstemcelltherapyautologousallogeneicorxenogeneic
    @2c.Isthestemcelllineagespecified @2d.Stemcellsource
    @2e.Aretransplantprocessingorqualityassuranceproceduresspecified @2f.
Transplantationprocedure
    @2g.Isimmunerejectionofthegraftaddressedinthetreatmentprotocol @2h.
Thetreatmentisportrayedasbeing
    @2i.Istherementionofclinicaltrialsconductedatclini @2j.Treatmentcost
    @2k.Ifcostfiguresorrangesareprovidedspecify @3a.Howthetreatmentworks
    @3b.StemcellsanddiseaseSiteportraysadeficiencyinafinitepoolofste
    @3c.Isthetreatmentisportrayedasrevolutionaryi.e.amarkeddeparture
    @3d.Isthetreatmentportrayedastheclinicalapplicationofroutineorfa
    @3e.Thesiteappealstoholisticnaturalormagicalpowersofrenewal
    @3f.Thesiteappealstothescientificdeconstructionofhealthandillnes
    @3g.Knowledgeisportrayedasbeingincompleteoruncertain
    @3h.Knowledgeisportrayedasbeingadvancedorcertain @3i.
Claimsaresubstantiatedmainlyby
    @4a.IndicationsAssociatedConditionsMannerofpresentation @4b.
Indeterminatevocabulary
    @4c.Associateddiseases @4d.Is“aging”anindication
    @4e.Arecosmeticorlifestyleenhancementpurposesgivenasindications
    @4f.Areanyconditionsorpatientsexcluded
    @5a.EfficacyoftreatmentDoeswebsitementiondiscussefficacyoftreatm
    @5b.Ifyesisanyevidenceforefficacyprovided @6a.
BenefitsoftreatmentMannerofpresentation
    @6b.Treatmentappearsgenerallybeneficial @6c.Associatedbenefitsinclude @6d.
Likelihoodofbenefits
    @7a.RisksoftreatmentMannerofpresentation @7b.Treatmentappearsgenerallysafe
    @7c.Associatedrisksinclude @7d.Likelihoodofrisks @8a.
EthicalissuesAresocialethicalissuesmentioned
    @8b.Relevancy @8c.Hastreatmentreceivedanyofficialapprovals
    @9a.WebsiteaestheticsandintendedaudienceThiswebsiteappearstotarg
```

```
    @9b.Websiteappeal–thewebsiteappealsmainlythroug@9c.Visualstyle–
thewebsiteismainly
    @9d.Language–thewebsite'slanguageismainly@9e.DisclaimersLegality–
thewebsiteprovides
    @10.Otherservicesavailableatclinic @11.
Pleaseprovideanyotherinformationthatmayseemrelevantorprovide
    @12.DoesthewebsitementionordiscussCanadianlawregulationsrelating
  /ORDER=ANALYSIS.
```

Frequencies

Notes

Output Created		12-JAN-2018 11:56:00
Comments		
Input	Data	C:\Users\uogbo\Dropbox\IMPACT GRANT\rescclinicstudycodingframe\Untitled2R.sav
	Active Dataset	DataSet1
	Filter	<none>
	Weight	<none>
	Split File	<none>
	N of Rows in Working Data File	15
Missing Value Handling	Definition of Missing	User-defined missing values are treated as missing.
	Cases Used	Statistics are based on all cases with valid data.

Notes

Syntax	FREQUENCIES<br>VARIABLES=Clinic# @1a.<br>Nameofoganization @1b.<br>WebsiteURL @1c.<br>Datewebsitecoded<br>@1d.<br>Primarymaincliniclocation<br>withinCanada @1e.<br>Whetherthecompanyhasac<br>onnectiontoaforeignprovid<br>er<br>@2a.Stemcellofferings<br>@2b.<br>Isstemcelltherapyautologo<br>usallogeneicorxenogeneic<br>@2c.<br>Isthestemcelllineagespecifi<br>ed @2d.Stemcellsource<br>@2e.<br>Aretransplantprocessingor<br>qualityassuranceprocedur<br>esspecified @2f.<br>Transplantationprocedure<br>@2g.<br>Isimmunerejectionofthegra<br>ftaddressedinthetreatment<br>protocol @2h.<br>Thetreatmentisportrayeda<br>sbeing<br>@2i.<br>Istherementionofclinicaltria<br>lsconductedatclinic @2j.<br>Treatmentcost<br>@2k.<br>Ifcostfiguresorrangesarepr<br>ovidedspecify @3a.<br>Howthetreatmentworks<br>@3b.<br>StemcellsanddiseaseSitep<br>ortraysadeficiencyinafinite<br>poolofste<br>@3c.<br>Isthetreatmentisportrayeda<br>srevolutionaryi.e.<br>amarkeddeparture<br>@3d.<br>Isthetreatmentportrayedas<br>theclinicalapplicationofrout<br>ineorfa<br>@3e.<br>Thesiteappealstoholisticna<br>turalormagicalpowersofren<br>ewal<br>@3f.<br>Thesiteappealstothescienti<br>ficdeconstructionofhealtha<br>ndillnes<br>@3g.<br>Knowledgeisportrayedasb<br>eingincompleteoruncertain<br>@3h.<br>[illegible]

Notes

Resources	Processor Time	00:00:00.08
	Elapsed Time	00:00:00.05

```
[DataSet1] C:\Users\uogbo\Dropbox\IMPACT GRANT\rescclinicstudycodingframe\Untitled2R.sav
```

Statistics

		Clinic #	1a. Name of oganization	1b. Website URL	1c. Date website coded	1d. Primary/main clinic location within Canada
N	Valid	15	15	15	15	15
	Missing	0	0	0	0	0

Statistics

		1e. Whether the company has a connection to a foreign provider	2a. Stem cell offerings	2b. Is stem cell therapy autologous, allogeneic, or xenogeneic?	2c. Is the stem cell lineage specified?	2d. Stem cell source
N	Valid	15	15	15	15	15
	Missing	0	0	0	0	0

Statistics

		2e. Are transplant processing or quality assurance procedures specified?	2f. Transplantation procedure	2g. Is immune rejection of the graft addressed in the treatment protocol?	2h. The treatment is portrayed as being	2i. Is there mention of clinical trials conducted at clinic?
N	Valid	15	15	15	15	15
	Missing	0	0	0	0	0

Statistics

		2j. Treatment cost	2k. If cost figures or ranges are provided, specify.	3a. How the treatment works	3b. Stem cells and disease: Site portrays a deficiency in a finite pool of stem cells as a major mechanism or contributor to disease.	3c. Is the treatment is portrayed as revolutionary, i. e.: a marked departure from current therapies?
N	Valid	15	0	15	15	15
	Missing	0	15	0	0	0

Statistics

		3d. Is the treatment portrayed as the clinical application of routine or familiar procedures?	3e. The site appeals to holistic, natural, or magical powers of renewal	3f. The site appeals to the scientific deconstruction of health and illness	3g. Knowledge is portrayed as being incomplete or uncertain	3h. Knowledge is portrayed as being advanced or certain
N	Valid	15	15	15	15	15
	Missing	0	0	0	0	0

Statistics

		3i. Claims are substantiated mainly by ...	4a. Indications/Associated Conditions - Manner of presentation	4b. Indeterminate vocabulary	4c. Associated diseases	4d. Is “aging” an indication?
N	Valid	15	15	15	15	15
	Missing	0	0	0	0	0

Statistics

		4e. Are cosmetic or lifestyle enhancement purposes given as indications?	4f. Are any conditions or patients excluded?	5a. Efficacy of treatment - Does website mention/discuss efficacy of treatment?	5b. If yes, is any evidence for efficacy provided?	6a. Benefits of treatment - Manner of presentation
N	Valid	15	15	15	15	15
	Missing	0	0	0	0	0

Statistics

		6b. Treatment appears generally beneficial	6c. Associated benefits include	6d. Likelihood of benefits	7a. Risks of treatment - Manner of presentation	7b. Treatment appears generally safe
N	Valid	15	15	15	15	15
	Missing	0	0	0	0	0

Statistics

		7c. Associated risks include	7d. Likelihood of risks	8a. Ethical issues - Are social/ethical issues mentioned?	8b. Relevancy	8c. Has treatment received any official approvals?
N	Valid	15	15	15	15	15
	Missing	0	0	0	0	0

Statistics

		9a. Website aesthetics and intended audience - This website appears to target	9b. Website appeal – the website appeals mainly through	9c. Visual style – the website is mainly ...	9d. Language – the website's language is mainly	9e. Disclaimers/ Legality – the website provides
N	Valid	15	15	15	15	15
	Missing	0	0	0	0	0

Statistics

		10. Other services available at clinic	11. Please provide any other information that may seem relevant, or provides quotations of language used.	12. Does the website mention or discuss Canadian law/regulations relating to stem cells, embryonic stem cells or iPSCs?
N	Valid	15	0	15
	Missing	0	15	0

Frequency Table

Clinic #

		Frequency	Percent	Valid Percent	Cumulative Percent
Valid	1	1	6.7	6.7	6.7
	2	1	6.7	6.7	13.3
	3	1	6.7	6.7	20.0
	4	1	6.7	6.7	26.7
	5	1	6.7	6.7	33.3
	6	1	6.7	6.7	40.0
	7	1	6.7	6.7	46.7
	8	1	6.7	6.7	53.3
	9	1	6.7	6.7	60.0
	10	1	6.7	6.7	66.7
	11	1	6.7	6.7	73.3
	12	1	6.7	6.7	80.0
	13	1	6.7	6.7	86.7
	14	1	6.7	6.7	93.3
	15	1	6.7	6.7	100.0
	Total	15	100.0	100.0	

1a. Name of oganization

		Frequency	Percent	Valid Percent	Cumulative Percent
Valid	CAPRI (Central Alberta Pain & Rehab Institute)	1	6.7	6.7	6.7
	Cleveland Clinic Canada	1	6.7	6.7	13.3
	Clinique du Docteur Sylvain Simard	1	6.7	6.7	20.0
	Dash & Reed Sport Medicine	1	6.7	6.7	26.7
	Global Healthcare Connections Inc.	1	6.7	6.7	33.3
	Hair Renewal Specialists	1	6.7	6.7	40.0
	Horizon Joint Rejuvenation Center	1	6.7	6.7	46.7
	Inovo Medical	1	6.7	6.7	53.3
	KOPI (Kingston Orthopaedic Pain Institute) Stem Cell	1	6.7	6.7	60.0
	Orthopaedic Sport Institute	1	6.7	6.7	66.7
	Pagdin Health	1	6.7	6.7	73.3
	Phoenix MedSpa	1	6.7	6.7	80.0
	Provincial Oral Surgery	1	6.7	6.7	86.7
	RegenaStem (BioLinc Brock University)	1	6.7	6.7	93.3
	SEMI (Sports & Exercise Medicine Institute)	1	6.7	6.7	100.0
	Total	15	100.0	100.0	

1b. Website URL

		Frequency	Percent	Valid Percent	Cumulative Percent
Valid	http://dashreed.ca/our-services/sport-medicine/	1	6.7	6.7	6.7
	http://inovomedical.ca/services/about-regenerative-medicine/tissue-renewal-with-stem-cell-injections	1	6.7	6.7	13.3
	http://www.capriclinic.ca/	1	6.7	6.7	20.0
	http://www.cellulessouches.ca/?lang=en	1	6.7	6.7	26.7
	http://www.drgrantpagdin.com/services/stem-cell-medicine/	1	6.7	6.7	33.3
	http://www.globalhealthcareconnections.com/html/procedures/Oncology-Cancer-Care-/Stem-Cell-Cancer-Treatment/index.cfm	1	6.7	6.7	40.0
	http://www.hairrenewalspecialists.com/how-we-do-it/	1	6.7	6.7	46.7
	http://www.phoenixmedspa.ca/stem-cell-therapy.php	1	6.7	6.7	53.3
	http://www.regenastem.com/company.html	1	6.7	6.7	60.0
	http://www.semisportmed.com/stem-cell-transplantation.html	1	6.7	6.7	66.7
	https://my.clevelandclinic.org/canada/services/prp-stem-cell-injections	1	6.7	6.7	73.3
	https://ortho-sport.ca/service/stem-cell-injection/	1	6.7	6.7	80.0
	https://provincialoralsurgery.com/stem-cells.php	1	6.7	6.7	86.7
	https://www.hjrcentre.com/	1	6.7	6.7	93.3

1b. Website URL

		Frequency	Percent	Valid Percent	Cumulative Percent
	https://www.kopistemcells.ca/	1	6.7	6.7	100.0
	Total	15	100.0	100.0	

1c. Date website coded

		Frequency	Percent	Valid Percent	Cumulative Percent
Valid	08/21/2017	3	20.0	20.0	20.0
	08/28/2017	1	6.7	6.7	26.7
	08/30/2017	2	13.3	13.3	40.0
	08/31/2017	3	20.0	20.0	60.0
	09/01/2017	6	40.0	40.0	100.0
	Total	15	100.0	100.0	

1d. Primary/main clinic location within Canada

		Frequency	Percent	Valid Percent	Cumulative Percent
Valid	1	1	6.7	6.7	6.7
	2	2	13.3	13.3	20.0
	3	3	20.0	20.0	40.0
	5	7	46.7	46.7	86.7
	6	1	6.7	6.7	93.3
	10	1	6.7	6.7	100.0
	Total	15	100.0	100.0	

1e. Whether the company has a connection to a foreign provider

		Frequency	Percent	Valid Percent	Cumulative Percent
Valid	0	10	66.7	66.7	66.7
	4	1	6.7	6.7	73.3
	5	1	6.7	6.7	80.0
	99	3	20.0	20.0	100.0
	Total	15	100.0	100.0	

2a. Stem cell offerings

		Frequency	Percent	Valid Percent	Cumulative Percent
Valid	0	2	13.3	13.3	13.3
	3	13	86.7	86.7	100.0
	Total	15	100.0	100.0	

2b. Is stem cell therapy autologous, allogeneic, or xenogeneic?

		Frequency	Percent	Valid Percent	Cumulative Percent
Valid	0	4	26.7	26.7	26.7
	1	11	73.3	73.3	100.0
	Total	15	100.0	100.0	

2c. Is the stem cell lineage specified?

		Frequency	Percent	Valid Percent	Cumulative Percent
Valid	0	5	33.3	33.3	33.3
	3	6	40.0	40.0	73.3
	4	4	26.7	26.7	100.0
	Total	15	100.0	100.0	

2d. Stem cell source

		Frequency	Percent	Valid Percent	Cumulative Percent
Valid	0	2	13.3	13.3	13.3
	1	3	20.0	20.0	33.3
	2	6	40.0	40.0	73.3
	5	1	6.7	6.7	80.0
	6	3	20.0	20.0	100.0
	Total	15	100.0	100.0	

2e. Are transplant processing or quality assurance procedures specified?

		Frequency	Percent	Valid Percent	Cumulative Percent
Valid	0	15	100.0	100.0	100.0

2f. Transplantation procedure

		Frequency	Percent	Valid Percent	Cumulative Percent
Valid	0	5	33.3	33.3	33.3
	1	1	6.7	6.7	40.0
	2	6	40.0	40.0	80.0
	5	3	20.0	20.0	100.0
	Total	15	100.0	100.0	

2g. Is immune rejection of the graft addressed in the treatment protocol?

		Frequency	Percent	Valid Percent	Cumulative Percent
Valid	0	15	100.0	100.0	100.0

2h. The treatment is portrayed as being

		Frequency	Percent	Valid Percent	Cumulative Percent
Valid	0	1	6.7	6.7	6.7
	1	1	6.7	6.7	13.3
	2	10	66.7	66.7	80.0
	3	3	20.0	20.0	100.0
	Total	15	100.0	100.0	

2i. Is there mention of clinical trials conducted at clinic?

		Frequency	Percent	Valid Percent	Cumulative Percent
Valid	0	15	100.0	100.0	100.0

2j. Treatment cost

		Frequency	Percent	Valid Percent	Cumulative Percent
Valid	0	14	93.3	93.3	93.3
	1	1	6.7	6.7	100.0
	Total	15	100.0	100.0	

2k. If cost figures or ranges are provided, specify.

		Frequency	Percent
Missing	System	15	100.0

3a. How the treatment works

		Frequency	Percent	Valid Percent	Cumulative Percent
Valid	0	7	46.7	46.7	46.7
	4	7	46.7	46.7	93.3
	5	1	6.7	6.7	100.0
	Total	15	100.0	100.0	

3b. Stem cells and disease: Site portrays a deficiency in a finite pool of stem cells as a major mechanism or contributor to disease.

		Frequency	Percent	Valid Percent	Cumulative Percent
Valid	1	15	100.0	100.0	100.0

3c. Is the treatment is portrayed as revolutionary, i.e.: a marked departure from current therapies?

		Frequency	Percent	Valid Percent	Cumulative Percent
Valid	1	1	6.7	6.7	6.7
	2	9	60.0	60.0	66.7
	3	5	33.3	33.3	100.0
	Total	15	100.0	100.0	

3d. Is the treatment portrayed as the clinical application of routine or familiar procedures?

		Frequency	Percent	Valid Percent	Cumulative Percent
Valid	1	1	6.7	6.7	6.7
	2	11	73.3	73.3	80.0
	3	3	20.0	20.0	100.0
	Total	15	100.0	100.0	

3e. The site appeals to holistic, natural, or magical powers of renewal

		Frequency	Percent	Valid Percent	Cumulative Percent
Valid	1	14	93.3	93.3	93.3
	2	1	6.7	6.7	100.0
	Total	15	100.0	100.0	

3f. The site appeals to the scientific deconstruction of health and illness

		Frequency	Percent	Valid Percent	Cumulative Percent
Valid	1	14	93.3	93.3	93.3
	2	1	6.7	6.7	100.0
	Total	15	100.0	100.0	

3g. Knowledge is portrayed as being incomplete or uncertain

		Frequency	Percent	Valid Percent	Cumulative Percent
Valid	1	10	66.7	66.7	66.7
	2	4	26.7	26.7	93.3
	3	1	6.7	6.7	100.0
	Total	15	100.0	100.0	

3h. Knowledge is portrayed as being advanced or certain

		Frequency	Percent	Valid Percent	Cumulative Percent
Valid	1	1	6.7	6.7	6.7
	2	11	73.3	73.3	80.0
	3	3	20.0	20.0	100.0
	Total	15	100.0	100.0	

3i. Claims are substantiated mainly by ...

		Frequency	Percent	Valid Percent	Cumulative Percent
Valid	0	8	53.3	53.3	53.3
	4	1	6.7	6.7	60.0
	6	3	20.0	20.0	80.0
	7	1	6.7	6.7	86.7
	10	2	13.3	13.3	100.0
	Total	15	100.0	100.0	

4a. Indications/Associated Conditions - Manner of presentation

		Frequency	Percent	Valid Percent	Cumulative Percent
Valid	0	6	40.0	40.0	40.0
	2	9	60.0	60.0	100.0
	Total	15	100.0	100.0	

4b. Indeterminate vocabulary

		Frequency	Percent	Valid Percent	Cumulative Percent
Valid	0	13	86.7	86.7	86.7
	1	2	13.3	13.3	100.0
	Total	15	100.0	100.0	

4c. Associated diseases

		Frequency	Percent	Valid Percent	Cumulative Percent
Valid	0	7	46.7	46.7	46.7
	6	5	33.3	33.3	80.0
	11	3	20.0	20.0	100.0
	Total	15	100.0	100.0	

4d. Is “aging” an indication?

		Frequency	Percent	Valid Percent	Cumulative Percent
Valid	0	13	86.7	86.7	86.7
	1	2	13.3	13.3	100.0
	Total	15	100.0	100.0	

4e. Are cosmetic or lifestyle enhancement purposes given as indications?

		Frequency	Percent	Valid Percent	Cumulative Percent
Valid	0	10	66.7	66.7	66.7
	1	2	13.3	13.3	80.0
	2	1	6.7	6.7	86.7
	3	2	13.3	13.3	100.0
	Total	15	100.0	100.0	

4f. Are any conditions or patients excluded?

		Frequency	Percent	Valid Percent	Cumulative Percent
Valid	0	12	80.0	80.0	80.0
	1	1	6.7	6.7	86.7
	4	2	13.3	13.3	100.0
	Total	15	100.0	100.0	

5a. Efficacy of treatment - Does website mention/discuss efficacy of treatment?

		Frequency	Percent	Valid Percent	Cumulative Percent
Valid	0	7	46.7	46.7	46.7
	1	8	53.3	53.3	100.0
	Total	15	100.0	100.0	

5b. If yes, is any evidence for efficacy provided?

		Frequency	Percent	Valid Percent	Cumulative Percent
Valid	0	3	20.0	20.0	20.0
	1	2	13.3	13.3	33.3
	2	2	13.3	13.3	46.7
	4	1	6.7	6.7	53.3
	99	7	46.7	46.7	100.0
	Total	15	100.0	100.0	

6a. Benefits of treatment - Manner of presentation

		Frequency	Percent	Valid Percent	Cumulative Percent
Valid	0	5	33.3	33.3	33.3
	1	1	6.7	6.7	40.0
	2	9	60.0	60.0	100.0
	Total	15	100.0	100.0	

6b. Treatment appears generally beneficial

		Frequency	Percent	Valid Percent	Cumulative Percent
Valid	1	12	80.0	80.0	80.0
	99	3	20.0	20.0	100.0
	Total	15	100.0	100.0	

6c. Associated benefits include

		Frequency	Percent	Valid Percent	Cumulative Percent
Valid	0	3	20.0	20.0	20.0
	1	10	66.7	66.7	86.7
	2	1	6.7	6.7	93.3
	4	1	6.7	6.7	100.0
	Total	15	100.0	100.0	

6d. Likelihood of benefits

		Frequency	Percent	Valid Percent	Cumulative Percent
Valid	0	3	20.0	20.0	20.0
	1	9	60.0	60.0	80.0
	4	2	13.3	13.3	93.3
	5	1	6.7	6.7	100.0
	Total	15	100.0	100.0	

7a. Risks of treatment - Manner of presentation

		Frequency	Percent	Valid Percent	Cumulative Percent
Valid	0	11	73.3	73.3	73.3
	1	1	6.7	6.7	80.0
	2	2	13.3	13.3	93.3
	3	1	6.7	6.7	100.0
	Total	15	100.0	100.0	

7b. Treatment appears generally safe

		Frequency	Percent	Valid Percent	Cumulative Percent
Valid	1	5	33.3	33.3	33.3
	99	10	66.7	66.7	100.0
	Total	15	100.0	100.0	

7c. Associated risks include

		Frequency	Percent	Valid Percent	Cumulative Percent
Valid	0	10	66.7	66.7	66.7
	3	1	6.7	6.7	73.3
	7	2	13.3	13.3	86.7
	8	1	6.7	6.7	93.3
	9	1	6.7	6.7	100.0
	Total	15	100.0	100.0	

7d. Likelihood of risks

		Frequency	Percent	Valid Percent	Cumulative Percent
Valid	0	10	66.7	66.7	66.7
	2	3	20.0	20.0	86.7
	4	2	13.3	13.3	100.0
	Total	15	100.0	100.0	

8a. Ethical issues - Are social/ethical issues mentioned?

		Frequency	Percent	Valid Percent	Cumulative Percent
Valid	0	11	73.3	73.3	73.3
	2	2	13.3	13.3	86.7
	3	2	13.3	13.3	100.0
	Total	15	100.0	100.0	

8b. Relevancy

		Frequency	Percent	Valid Percent	Cumulative Percent
Valid	0	12	80.0	80.0	80.0
	1	1	6.7	6.7	86.7
	3	2	13.3	13.3	100.0
	Total	15	100.0	100.0	

8c. Has treatment received any official approvals?

		Frequency	Percent	Valid Percent	Cumulative Percent
Valid	0	14	93.3	93.3	93.3
	2	1	6.7	6.7	100.0
	Total	15	100.0	100.0	

9a. Website aesthetics and intended audience - This website appears to target

		Frequency	Percent	Valid Percent	Cumulative Percent
Valid	1	11	73.3	73.3	73.3
	3	1	6.7	6.7	80.0
	6	3	20.0	20.0	100.0
	Total	15	100.0	100.0	

9b. Website appeal – the website appeals mainly through

		Frequency	Percent	Valid Percent	Cumulative Percent
Valid	1	1	6.7	6.7	6.7
	4	2	13.3	13.3	20.0
	5	9	60.0	60.0	80.0
	8	1	6.7	6.7	86.7
	9	1	6.7	6.7	93.3
	10	1	6.7	6.7	100.0
	Total	15	100.0	100.0	

9c. Visual style – the website is mainly ...

		Frequency	Percent	Valid Percent	Cumulative Percent
Valid	1	3	20.0	20.0	20.0
	2	12	80.0	80.0	100.0
	Total	15	100.0	100.0	

9d. Language – the website's language is mainly

		Frequency	Percent	Valid Percent	Cumulative Percent
Valid	1	14	93.3	93.3	93.3
	4	1	6.7	6.7	100.0
	Total	15	100.0	100.0	

9e. Disclaimers/ Legality – the website provides

		Frequency	Percent	Valid Percent	Cumulative Percent
Valid	0	10	66.7	66.7	66.7
	1	1	6.7	6.7	73.3
	3	1	6.7	6.7	80.0
	5	3	20.0	20.0	100.0
	Total	15	100.0	100.0	

10. Other services available at clinic

		Frequency	Percent	Valid Percent	Cumulative Percent
Valid	0	2	13.3	13.3	13.3
	9	1	6.7	6.7	20.0
	10	12	80.0	80.0	100.0
	Total	15	100.0	100.0	

11. Please provide any other information that may seem relevant, or provides quotations of language used.

		Frequency	Percent
Missing	System	15	100.0

12. Does the website mention or discuss Canadian law/regulations relating to stem cells, embryonic stem cells or iPSCs?

		Frequency	Percent	Valid Percent	Cumulative Percent
Valid	0	12	80.0	80.0	80.0
	1	3	20.0	20.0	100.0
	Total	15	100.0	100.0	
